# Understanding sciatica: illness and treatment beliefs in a lumbar radicular pain population. A qualitative interview study

**DOI:** 10.3399/bjgpopen19X101654

**Published:** 2019-08-07

**Authors:** Robert Goldsmith, Nefyn Howard Williams, Fiona Wood

**Affiliations:** 1 Advanced Physiotherapy Practitioner, Bangor University and Cardiff and Vale University Health Board, Cardiff, UK; 2 Professor in Primary Care, University of Liverpool, Liverpool, UK; 3 Reader, Division of Population Medicine, Cardiff University, Cardiff, UK

**Keywords:** Sciatica, Low back pain, Radicular pain, Illness beliefs, Qualitative research, Primary health care, Treatment beliefs

## Abstract

**Background:**

Several pathological processes contribute to lumbar radicular pain (LRP), commonly known as sciatica. It is not known how patients rationalise the experience of sciatica or understand the diagnosis. Providing clinicians with a better understanding of how patients conceptualise sciatica will help them to tailor information for patients on the management and treatment of the condition.

**Aim:**

To understand patients’ beliefs regarding their illness following a diagnosis of LRP, how these beliefs were developed, and the impact of illness beliefs on treatment beliefs.

**Design & setting:**

Qualitative interview study from a single NHS musculoskeletal interface service (in Wales, UK).

**Method:**

Thirteen patients recently diagnosed with LRP were consecutively recruited. Individual semi-structured interviews were recorded and transcribed. Data were analysed using a thematic approach.

**Results:**

Four main themes were generated: (1) the illness experience (2) the concept of sciatica, (3) treatment beliefs, and (4) the desire for credible information.

**Conclusion:**

The diagnosis of LRP is often communicated and understood within a compressive conceptual illness identity. Explaining symptoms with a compressive pathological model is easily understood by patients but may not accurately reflect the spectrum of pathological processes known to contribute to radicular pain. This model appears to inform patient beliefs about treatments. Clinicians should take care to fully explain the pathology prior to shared decision-making with patients.

## How this fits in

LRP (sciatica) is often explained and understood as being caused by nerve compression. While this is often part of the pathological process, inflammation and neural sensitisation play important roles. Explaining radicular pain in terms of compression alone may influence treatment decisions and leave patients with no evidence of compression without an explanation for their pain. Clinicians should take care to include the roles of inflammation and sensitisation within their discussion of radicular pain pathology.

## Introduction

Commonly known as ‘sciatica’, LRP is a painful and disabling condition, resulting in worse pain, disability, and quality of life, and increased use of health resources compared with low back pain alone.^1^

Repeated findings have challenged the adequacy of the pathoanatomical model to fully explain LRP pathology. Although LRP is often caused by an intervertebral disc prolapse,^2^ nerve root compression is commonly seen in asymptomatic populations.^3^ Serial imaging is only able to demonstrate a ‘new’ compression in 3%–7% of incident radicular symptoms.^4^ Furthermore, the degree of neural compression does not correlate with symptoms^5^ or recovery.^6,7^ Approximately 67% of disc herniations will spontaneously reabsorb, but symptoms may persist.^8^

Although LRP can arise from mechanical compression of the nerve root (causing hypoxic damage), the presence of pro-inflammatory material from the disc is required to sensitise nerve roots.^9,10^ Mechanical stimulation of nerve roots that have not been exposed to inflammation does not cause radicular pain.^11^ Conversely, radicular pain has been observed without compression.^12,13^ There is increasing evidence that radicular pain may involve almost all types of pain, including ischaemic, inflammatory, mechanical, and neuropathic pain.^14^

Despite this complexity, the natural history of LRP is generally considered to be favourable, with estimates of 60% achieving a good outcome (≥30% improvement in disability) by 3 months and 55%–70% by 1 year.^7,15^ During this time period, there can be significant impact on the individual, with LRP perceived to be ‘constant, intense, and all-encompassing’.^16^

Ong *et al* ^16^ explored patient beliefs in a ‘sciatica’ population. The identification of a cause (such as an event or behaviour) and the use of medical terms to describe and legitimise their pain were important to people because they provided an explanation of why they were in pain.^16^

The Common Sense Model (CSM) of self-regulation^17^ is a framework for understanding illness perceptions. The CSM explores patient perceptions of cause, prognosis, consequences, and treatment effectiveness. The model assumes that as people obtain new information about their condition and evaluate their attempts to moderate or cope with its effects, illness representations are formed and thereafter continually refined.^17^ The process of refining illness representations is influenced by personal experience as well as the opinions of significant others, health professionals, and media sources.^18,19^ Illness representations have been correlated with maladaptive coping, self-efficacy, anxiety, depression, and quality of life in other clinical populations.^20,21^ The CSM assumes that people aim for coherence between illness representations and treatment beliefs, matching treatment decisions to their illness.^22^ Previous reviews have recommended that researchers consider the combined role of both illness and treatment beliefs in physical illnesses.^23^

The aim of this study was to understand patients’ conceptual beliefs of sciatica and what might influence their development, using the illness identity component of the CSM. The authors sought to understand how illness representations might influence perceptions of different treatment options.

## Method

### Participants and recruitment

The setting was a musculoskeletal triage service in a Welsh NHS health board serving a mixed urban and suburban adult population of approximately 400 000 people.^24^ Participants were referred to the service by their GP, and were aged ≥18 years, with symptoms for more than 4 weeks and a clinical diagnosis of radicular pain (Box 1). After their usual care, eligible candidates were approached by their clinician, an advanced physiotherapy practitioner. Participants were consecutively sampled for a homogenous diagnosis according to the criteria for LRP,^25,26^ between January–September 2018. A total of 36 recruitment packs were given out. Reasons for non-participation are not known. Of those participants who responded to the recruitment information, one was excluded (due to bilateral leg pain), and the remaining 13 participants completed the study.

Box 1 **Criteria for lumbar radicular pain^25,26^**

**Table:** 

Symptoms duration >4 weeks Unilateral leg pain radiating below the knee Leg pain worse than, or as bad as, back pain (if present) Positive straight leg raise test OR muscle weakness in one myotome OR loss of sensation in one dermatome No pain in contralateral leg below the gluteal margin

Participants were excluded if they reported bilateral leg pain, cauda equina symptoms, or there was clinical suspicion of sinister pathology. Additional exclusion criteria included inability to speak English, a lack of capacity for informed consent, or any prior contact with the researcher. Potential participants were given written information regarding the study and asked to return a reply slip to the research team if they were interested in contributing.

### Data collection

An interview guide was developed using items from the Illness Perception Questionnaire (IPQ)^27^ and Low Back Pain Treatment Beliefs Questionnaire (LBP-TBQ);^22^ further information is available from the authors on request. Open questions were used to prompt reflection and discussion, and the interviewer reflexively explored emergent issues as they developed.

Care was taken to minimise researcher bias throughout the data collection and analysis cycle. The interviewer (a male advanced physiotherapy practitioner with >10 years clinical experience, and an MSc in Physiotherapy), received 3 days of specialist training in conducting qualitative interviews. The research team also included a GP and social scientist, which ensured a range of perspectives and helped the researchers check each other’s assumptions. Prior to data collection, the interviewer documented his assumptions and biases regarding radicular pathology and its management (these included a belief in the importance of patient education, shared decision-making, and the limitations of magnetic resonance imaging [MRI] findings to explain symptoms). Three practice interviews were also conducted, and feedback was sought. The interviewer identified himself to participants as a researcher who was interested in patients’ understanding of their condition.

To put participants at ease, the one-to-one interviews were conducted either in the participant’s own home or in a quiet room within a hospital, separated from the physiotherapy department (participants chose which location).

Individual, in-depth, face-to-face interviews were audiorecorded and transcribed verbatim. Participants chose pseudonyms to maintain anonymity. In order to minimise bias and maximise the richness of responses, field notes were kept reflecting on interview performance and style. Analytical memos of emergent content were recorded.

To inform judgments of transferability, participants completed a demographic questionnaire, the Tampa Scale of Kinesiophobia,^28^ and the Modified Rolland Morris Disability Scale^29^ before the interview. The questionnaires were chosen as illness beliefs have been associated with disability and fear of movement.^30,31^

Recruitment was continuously reviewed for information power and level of saturation by the lead author, in discussion with other team members. A consensus judgment that sufficient saturation had taken place was achieved when data were available for each of the nine initial a priori codes, and no new codes or themes were emerging from the data. After the 11th interview, the interviewer felt that he was hearing repetition of previous data with no new insights or themes being developed (informational redundancy).^32^ Data from two further interviews were collected, and it was confirmed that no new themes were emerging. Although the sample size may appear relatively modest, the interviews were found to have significant ‘information power’, sufficient to allow in-depth analysis.^33^

### Data analysis

The lead author familiarised himself with the data before initially coding all interviews using NVivo (version 12) software. In order to achieve a balance between within- and across-case analysis, emergent themes were identified and reviewed using a template analysis approach.^34^ An initial a priori coding template was developed using terms derived from the illness dimensions of the IPQ (*identity, consequences, timeline, control/cure* and *cause*), and the treatment dimensions from the LBP-TBQ (*credibility, effectiveness, concerns*, and *individual ‘fit*’). This coding framework was refined during the analysis, with a priori codes being redefined, deleted, or replaced with codes emerging from the data to form a final coding template. To ensure the analysis remained sensitive to uncovering the beliefs, attitudes, and values of individual participants, a ‘one sheet of paper’ analysis^35^ for each participant was performed to create additional conceptual themes and contexts. Themes were continually reviewed and refined throughout analysis. Member-checking and participant feedback on themes were not used as it was considered too onerous on the participants.

## Results

Thirteen participants were recruited, aged 21–76 years (median 53 years), with symptom duration ranging from 4 months to 40 years (median 9 months). Full participant characteristics are described in Table 1. The interview duration ranged from 33–64 minutes (median 45 minutes). Results include illustrative quotes with pseudonyms.

**Table 1.** Participant characteristics

Alias, age, sex	Occupation and work status	Symptoms	Duration	Course	Imaging	Treatments received	RMDQ	TSK	Management plan
Edward 71, M	Retired	Leg pain	40 years	Episodic (not in an episode at the time of interview)	MRI	Medication, acupuncture, chiropractic, physiotherapy	1/24	32	Self-management
Myfanwy, 53, F	Police officer; on sick leave due to symptoms	Leg pain > back pain	8 months	Episodic	MRI	Medication, chiropractic, physiotherapy, spinal injection	11/24	41	Review with APP
Gordon, 57, M	Delivery driver; unemployed; stopped work due to symptoms	Leg pain	10 months	Severe, constant	XRMRI	Medication	22/24	43	Physiotherapy
Gethin, 42, M	Employed	Leg pain> back pain, foot numbness	1 year	Constant, not changing	MRI	Osteopathy	14/24	33	Physiotherapy and review with consultant
Marley, 52, M	Self-employed, runs own training company	Leg pain	3 years	Constant, variable	MRI	Medication, osteopathy, physiotherapy, spinal surgery	3/24	23	Nerve root block±surgical opinion
Ricardo, 45, M	Engineer;occasional days off	Leg pain > back pain, foot numbness	2 years	Deteriorating	MRI	Medication, acupuncture, physiotherapy	16/24	48	Surgical opinion
Margret, 76, F	Retired	Leg pain	3 months	Improving	XR	Medication, physiotherapy	5/24	32	Self-management
Maria, 63, F	Employed carer; off work 3 months	Leg pain > back pain	9 months	Variable, but improving overall	XRMRI	Medication, acupuncture, chiropractic, physiotherapy	17/24	45	Nerve root block
Megan, 21, F	Student and works in a supermarket	Leg pain	8 months	Severe, constant, deteriorating	MRI	Medication, chiropractic, physiotherapy	20/24	43	Surgical opinion
Harry, 44, M	Civil servant; intermittent time off	Leg pain	18 years	Episodic, deteriorating	MRI	Medication, chiropractic, physiotherapy, nerve root block	18/24	45	Review with consultant
Patricia, 68, F	Retired	Leg pain and numbness	5 months	Improving	XRMRI	Medication, physiotherapy	8/24	42	Review with APP
Martha, 54, F	Teaching assistant; returned on light duties	Leg pain, foot cramps, numbness, and paraesthesia	4 months	Improving	None	Medication, osteopathy, physiotherapy	10/24	38	Physiotherapy
Hannah, 45, F	Healthcare support worker; returned on reduced hours	Leg pain, foot paraesthesia, and cramps	9 months	Severe, constant, not changing	MRI	Medication, massage therapy, physiotherapy	15/24	51	Nerve root block

APP = advanced physiotherapy practitioner. MRI = magnetic resonance imaging. RMDQ = Modified Rolland Morris Disability Questionnaire.^29^ TSK = Tampa Scale of Kinesiophobia (>37 high score, and ≤37 low scores).^28^ XR = X-ray.

‘>’ indicates patient’s relative rating of pain; for example, leg pain rated to be greater than back pain.

### Summary

Four major themes were generated: (1) the illness experience, (2) the concept of sciatica, (3) treatment beliefs, and (4) the desire for credible information (see Box 2 for summary).

Box 2 **Participant themes and subthemes**

**Table:** 

Theme	Subtheme within narrative
Illness experience	Severe, unpredictable pain Mentally and physically draining Pain not ‘visible’
Concept of sciatica	Nerves ‘tapped’ or ‘rubbed’ by a ‘swollen’ disc Confusion over role of posture, alignment, and leg length A disc might ‘shrink’ with time Compression needs to be resolved for pain to improve Confirmation of compression on MRI gives ‘validity’ to suffering Absence of compression results in frustration and a lack of validation
Treatment beliefs	*Exercise*: Helpful to ‘cope’, but does not address compression Weightlifting, bending, and impact might increase size of the disc or prevent it ‘shrinking’ Strength, swimming, yoga, stretching, and cycling improves 'health' and ‘protects’ the spine Warm-up improves elasticity of nerve and spinal tissues Stretching 'loosens up’ the nerve, or ‘pulls it clear' *Manual therapies*: ‘Realigns’ the spine, ‘frees up’ nerves, but does not address compression Massage helpful in managing symptoms Acupuncture possibly helpful, but not credible *Medication and nerve root block:* Temporary treatments, not a ‘fix’ Nerve root block works by ‘shrinking’ the disc, or improving the nerve ‘route’ *Surgery:* A powerful, definitive treatment; ‘fixes’ compression Higher risk
Desire for credible information	Seeking of information from a variety of sources Concern over credibility of information Clear explanations were highly valued Using a plastic model and MRI ‘explains’ the compression

#### Theme 1: The illness experience

Participants emphasised the severity and unpredictability of the pain experience, which was ‘mentally and physically draining’:

*‘ … it was absolutely excruciating. I felt like ten knives were being rammed into my body. It was horrendous. It took me completely by surprise.’*

(Maria, 63, F)

Three participants expressed suicidal thoughts:

*‘* […] *it’s like I’m constantly in excruciating pain. The idea of living to old age in this level of pain almost feels like it’s not an option. I’ve had, I guess you could consider, suicidal thoughts.’*

(Megan, 21, F)

Participants felt like their pain was not ‘visible’ and therefore not taken seriously:

*‘I feel like people have got no understanding of just how painful it can be. I don’t think it’s taken seriously enough. I don’t think people release just how painful it is. It is excruciating pain, constant.’*

(Maria, 63, F)

#### Theme 2: The concept of ‘sciatica’

All participants described a concept of ‘sciatica’ in terms of discs being ‘out of place’ or ‘swollen’, causing either ‘trapping’ or ‘rubbing’ of sensitive neural tissue:

*‘* […] *it’s getting fouled somehow. It’s getting rubbed. Aggravated. I don’t necessarily think it’s gonna be trapped, it’s being irritated* […] *’*

(Gordon, 57, M)

If inflammation was considered, it was in terms of ‘swelling’, occupying more space and contributing to compression. No participant reported incorporating ischaemic mechanisms within their illness construct:

*‘The way I understand it is my disc is inflamed so it’s bulging, so it’s bigger than normal, and that’s why it’s pressing.’*

(Gethin, 42, M)

Only one participant conceptualised inflammation as sensitising neural tissue. This participant had discussed his diagnosis with a close friend who was a physiotherapist working in elite sport. This suggests that credible information was capable of refining the illness construct for this individual:

*“I believe there was two things happening: one, the actual pressure of the nerve itself, but two, it was a chemical reaction* […] *those chemicals* […] *they have a burning effect, corrosive effect, and it can be very damaging.”*

(Marley, 52, M)

There was confusion over the role of posture, spinal alignment, and leg length, with participants receiving conflicting explanations:

*‘A sports masseur I went to, to check my alignment. He said my alignments were fine. And the physio told me my alignments were out. So, I kind of* … *I don’t think anybody really knows what they’re doing, to be honest.’*

(Hannah, 45, F)

Participants were aware of the possibility of a disc reducing in size with time, but were not sure if it would for them, how long this would take, or how this process takes place:

*‘I’m not sure you can change it, once a disc slipped. I don’t know. I’m assuming you can’t. I’ve read that they will eventually dry or shrivel or something and move back off the nerve.’*

(Maria, 63, F)

Many participants were aware of the potential for improvements in both the size of a disc and in their pain, with improvement or deterioration in pain being determined by a corresponding change in compression:

*‘The discs are abnormal and they’re bulging* […] *if they’re bulging, surely there’s got to be a way of getting them back to normal and when they’re back to normal I won’t have that pain.’*

(Megan, 21, F)

When an MRI confirmed a diagnosis, participants appreciated the scan making the pain ‘visible’, the apparent certainty of knowing what was causing their pain and the anticipation of treatment.

*‘I feel like at least I’ve got an answer to the pain… it was a bit of a relief knowing that at least … they know the problem now, that something can be done about it.’*

(Megan, 21, F)

When an MRI did not explain their symptoms, participants were left confused, frustrated, and wanting an explanation and validation of their painful experience:

*‘Why is one person having that pain, but the next person is quite happily skipping around with … with no symptoms at all? It doesn’t make sense.’*

(Myfanwy, 53, F)

Many were disappointed that the investigation had not identified a clear solution to their suffering, and participants with episodic symptoms felt it was important to have an MRI during an episode to detect the change in shape of the disc. When clinicians explained MRI findings by comparing to published asymptomatic MRI data, some participants were left with conflicting thoughts:

*‘You’re told that … lots of people have a very similar picture and don’t have any symptoms whatsoever. It’s frustrating because, if you’re in as much pain, it’s your body telling you there’s something very wrong and you need to adapt to it, because it’s a protective mechanism. Um, so it’s sort of fighting against the two ideas.’*

(Myfanwy, 53, F)

Participants appreciated the reassuring explanations they were given, even if they were not explained in terms that were easy to understand:

*‘I found some of the images quite difficult to follow. I didn’t want to ask too many questions. She made it seem that it wasn’t that serious, so I took her word for it. It was her tone of voice, really.’*

(Patricia, 68, F)

While participants were constructing a concept of sciatica, healthcare practitioners seemed to have been influential in its development, often using a plastic model of the spine:

*‘You’ve got the model of vertebrae and things like that … they were explaining that you’ve got your shock absorbers in-between the vertebrae there and mine are gone at the bottom, so I’ve got bone-on-bone which is trapping nerves and parts of my shock absorber stick out where they shouldn’t be, so they then hit the nerves* […] *’*

(Harry, 44, M)

#### Theme 3: The link between illness beliefs and treatment beliefs

##### Exercise

Exercise such as swimming, yoga, stretching, and cycling was viewed as being helpful to improve spinal 'health' and to ‘cope’ with symptoms, but not capable of changing the compression. There was a desire for a well-structured rehabilitation programme which was flexible enough to deliver individualised treatment in a group exercise setting and was overseen by a ‘credible’ person. Participants wanted a clear plan for support if exercises caused an increase in pain. Individualised rehabilitation was seen as important as some participants could not tolerate sitting on a cycling seat, some could not sit down in a chair for long, and others could not stand for long. There was a concern that weightlifting or repetitive bending and/or impact might increase size of the disc or prevent it ‘shrinking’:

*‘I should imagine a lot of exercise … would be unsafe. You’re in danger of making things worse. I wouldn’t want to push that disc any further than it is. I’m hoping it’s going back or shrivelling up.’*

(Maria, 63, F)

Several participants had been advised against running by healthcare practitioners. Conversely, strengthening the spine was ‘protective’ of future episodes. Adequate warm-up was perceived to improve the elasticity of nerve and surrounding spinal tissues, with stretching afterwards helpful to 'loosen up’ the nerve, or ‘pull it clear' from the compression:

*‘Well, if you’ve got something that’s slack, there’s more chance it’s gonna' foul' on something. I look at it like a piece of rope and if you pull that tight, hopefully it’ll make it clear.’*

(Gordon, 57, M)

##### Manual therapies

Participants reported that manual treatment was explained by practitioners as working via ‘realigning’ the spine and ‘freeing up’ nerves, but participants felt it was not capable of changing the cause of the compression:

*‘First of all, I saw a chiropractor because I thought there was gonna be a simple solution of some form of manipulation that was gonna, sort of, realign whatever was going on.’*

(Gethin, 42, M)

Participants felt that soft-tissue massage was helpful in managing symptoms, and acupuncture might be helpful, but lacked credibility.

##### Medication

While there was a strong desire for effective pain control, participants expressed concerns over effectiveness, side effects, and the potential for addiction with medications. Participants viewed medication as a short-term treatment that did not address the underlying cause:

*‘* […] *the ibuprofen was never gonna work on a bit of my disc protruding into that area, how I was hoping it was gonna do anything about that, I’m not so sure* […] *Don’t put a plaster on it, just get in there and sort this.’*

(Harry, 44, M)

##### Nerve root block

A nerve root block (NRB) was also seen as a short-term treatment option that worked by reducing compression:

*‘I’m assuming the injection would reduce the inflammation* […] *then it would reduce it* [the disc] *to the point that it’s not touching on the nerve* […] *but my view on it was once that wears off, the bulge will start coming back and then it’ll go back into it.’*

(Gethin, 42, M)

There was considerable uncertainty and confusion about how a nerve root injection might work. One participant misunderstood the term ‘root’ to mean ‘route’, which had not been clarified, and this impacted on how they viewed its mechanism of action:

*‘It’s a cortisone injection, route* [sic] *nerve block. By those words, I’ve got a nerve and there’s a route going down there and it gets blocked* […] *and that’s gonna try and take the spasm or swelling or anything that’s around my disc, and hopefully that should make the whole route clearer.’*

(Marley, 52, M)

##### Surgery

Surgery was perceived as involving higher risks, but was seen as taking decisive, definitive action that addressed the underlying problem:

*‘The way it’s been explained to me is that* […] *the discs are abnormal and they’re bulging. So that if they’re bulging, surely there’s got to be a way of getting them back to normal and when they’re back to normal I won’t have that pain* […] *’*

(Megan, 21, F)

*‘The process of the disc damage. I think once you’ve got it, I think the operation is the only solution.’*

(Harry, 44, M)

#### Theme 4: The desire for credible information

Explanations for their pain were highly valued by participants, and there was a desire for early access to credible sources of information to conceptualise their illness:

*‘* […] *if I just know myself what’s going on* […] *, I could probably deal with it myself just as effectively as taking half a day off work to go and sit at the GP surgery to get given ibuprofen or some other tranquiliser that I don’t really want to take.’*

(Harry, 44, M)

As well as asking healthcare practitioners, participants also searched for information from other sources (such as books, newpapers, social media, websites, and forums). There was a concern that the search for information could easily become obsessive and incorrect information could be unhelpful. When searching for information, participants were mindful of conflicting commercial interests and unrealistic expectations, but remained curious:

*‘I really found it difficult to access* [websites] *that I thought, “They’re not trying to sell me something here.”’*

(Harry, 44, M)

Several participants suggested ways to improve access to credible information for patients:

*‘If there is a video on sciatica and what exactly it is and how it’s caused, surely a short video or something like this, go to YouTube* […] *, you could learn everything, exactly what’s going on.’*

(Marley, 52, M)

## Discussion

### Summary

This study explored how people rationalise their painful experiences after being diagnosed with LRP. This is the first study to explore illness representations in this population. Four main themes were identified which describe the development of an explanatory construct: (1) the devastating experience requiring an explanation; (2) the illness identities that people construct; (3) the impact of the illness identity on treatment beliefs; and 4) an ongoing search for further credible information to refine their illness identity.

### Strengths and limitations

Participants were recruited from a single site, thus limiting transferability, although findings are consistent with previously published data in this field.

The unavoidable influence of the researcher’s position on the data collected is acknowledged. Several steps were taken to maintain reflexivity and limit bias throughout data collection (as detailed in the Method section). The use of a single analyst and data coder was mitigated by regular discussion with the supervisory team to ensure transparency and integrity of the analysis.

### Comparisons with existing literature

Consistent with previous studies, participants reported a range of symptoms that were physically and mentally draining,^16^ and feeling like they were not taken seriously^36^ as their symptoms were underappreciated, illegitimate, and invisible.

Three participants expressed current or previous suicidal ideation, which confirms previous findings.^36^ The unpredictability of sudden and severe pain episodes led to feelings of anxiety, dread, and despair, which is consistent with experimental evidence.^37^

Participants valued clear information on their diagnosis and appreciated the use of plastic models of the spine to help explanations. As with previous findings,^38^ this study found participants also highly valued clinicians taking the time to show them their MRI scan. It is likely that the use of plastic models and visualisation of MRI will have influenced the illness construct of participants. A diagnosis helped participants to either manage ongoing symptoms with a sense of certainty, or to look forward to a definitive treatment. Similar to previous studies, there was a desire for a clear prognosis.^16,38^ In contrast to previous work,^38^ this study did not find concern of serious pathology (such as malignancy).

In agreement with previous findings,^38^ this study found that when an MRI failed to adequately explain their experience, participants were left feeling invalidated, frustrated, and confused. The absence of compression on MRI was in direct conflict with their existing illness identity, leading to a distressing and confusing process of trying to construct a new one. None of these participants had been able to construct new meaningful identities to explain their ongoing symptoms.

The illness constructs appeared to have influenced beliefs regarding treatment effectiveness. Treatments addressing compression (such as surgery) were easily understood and perceived as having more control over the condition. Those treatments seeking to address inflammatory or neuropathic mechanisms (such as medication or NRB) were confusing and therefore perceived as less powerful. Despite being perceived as lacking control over compression, there was a clear desire for structured conservative management.

Participants expressed a desire for credible information as it was an important step in understanding their illness before considering treatment. As well as information from healthcare clinicians and social networks, participants gathered information from a variety of written media sources including newspapers, online forums, social media, and textbooks. Much of this information was from commercial companies advertising products and services. Some participants expressed concern over the credibility of the information, and scepticism of conflicting commercial interests, but were not clear how to select credible sources. Participants expressed a preference for visual, online information to support their decision-making.

### Implications for research and practice

These findings highlight the importance of asking patients to explain their understanding of LRP. Clinicians should to consider providing broader explanations for LRP pathology that include inflammatory and neuropathic pain mechanisms (which appeared to be lacking). Clinicians should take care to include these mechanisms when explaining a diagnosis using plastic models of the spine. This may be especially important when a compressive explanation is not conclusively demonstrated by MRI, as it will enable clinicians to validate and legitimise patients’ symptoms. The authors recommend caution when comparing MRIs of symptomatic versus asymptomatic patients, as this may have unintended delegitimising effects. They also agree with previous calls for accurate information-sharing regarding prognosis and prompt access to treatment, including psychological support.

Further research is required to understand the most effective type of interventions that support the development of accurate conceptual models for LRP. For example, including ischaemic mechanisms as part of an explanation of pathology may broaden a patient’s understanding, but the effect on behaviour and decision-making is unknown. Identifying the optimal content, context, and timing of interventions is likely to be important. Researchers should consider important outcomes such as patient satisfaction with decision-making, pain self-efficacy, and treatment expectations.

This is the first study to specifically explore illness constructs in an LRP population. It has confirmed the overwhelming impact of LRP, which drives the individual to construct an illness identity to conceptualise their suffering. Illness identities were firmly entrenched in a mechanical-compressive model that did not incorporate inflammatory, ischaemic, and neuropathic pain mechanisms. Consistent with the CSM, information from a variety of sources appeared to become interwoven with advice from healthcare clinicians to form and refine these illness identities. These findings have identified several opportunities to help patients construct more accurate illness identities, providing validation to those who need it and a clear foundation for shared decision-making.
